# Public Practices and Personal Perspectives

**DOI:** 10.1002/hast.709

**Published:** 2017-05-22

**Authors:** Gregory E. Kaebnick

**Affiliations:** ^1^ Hastings Center Report

## Abstract

*I once heard John Arras, who was one of bioethics’ bright lights and, toward the end of his life, a member of the Presidential Commission for the Study of Bioethical Issues, remark that it is hard for an ethics commission not to “do paint‐by‐numbers ethics.” What I think Arras had in mind is an approach that, in the set of essays that make up this special report, Rebecca Dresser describes as a listing of “general, often relatively uncontroversial” moral positions to support largely procedural recommendations. Arras was calling attention to one of the challenges and sometimes frustrations of commission thinking. It is a recurring topic in this special report, Goals and Practices of Public Bioethics, which features a series of reflections about how national bioethics commissions around the world have contributed to public understanding and public policy about bioethical issues. Both the topic and the authors are drawn from the final two public meetings of the PCSBI, which was the most recent U.S. example of a national bioethics commission and whose winding down created an occasion for pondering the different forms and functions of bioethics commissions*.

## Editor's Foreword

I once heard John Arras, who was one of bioethics’ bright lights and, toward the end of his life, a member of the Presidential Commission for the Study of Bioethical Issues, remark that it is hard for an ethics commission not to “do paint‐by‐numbers ethics.” What I think Arras had in mind is an approach that, in the set of essays that make up this special report, Rebecca Dresser describes as a listing of “general, often relatively uncontroversial” moral positions to support largely procedural recommendations. One advantage of general, uncontroversial moral positions is that they are the ones that commission members can actually agree on. Procedural recommendations, meanwhile, offer the advantage that they allow finer substantive points to be set aside until later and fine‐tuned to the demands of whatever case is then at hand. So—promote human welfare and, to the extent possible, let people affected by a decision determine what “promoting welfare” requires, in so doing exercising their autonomy. Amen.

Still, as Dresser notes, the result can be a “bland and anemic bioethics.” If a commission does not somehow dig deeper and get into more contested and less clear moral positions, it may be missing many important moral concerns. Morality also sometimes involves difficult concepts that are not universally shared, that are variously understood, that stand in tension with each other, and that can be carried out only with careful attention to social context.

Arras's comment was not meant as a reflection on whether the Presidential Commission for the Study of Bioethical Issues fell into “paint‐by‐numbers” ethics, and essays in this report by other PCSBI members—especially those by chair Amy Gutmann and vice chair James Wagner and by Daniel Sulmasy, about the commission's style of deliberation—show just how hard the members worked to pay thorough attention to the full array of concerns that they perceived in a problem and that the people who spoke at bioethics commission meetings expressed. But Arras was certainly calling attention to one of the challenges and sometimes frustrations of commission thinking. It is a recurring topic in this special report, which features a series of reflections about how national bioethics commissions around the world have contributed to public understanding and public policy about bioethical issues. Both the topic and the authors are drawn from the final two public meetings of the PCSBI, which was the most recent U.S. example of a national bioethics commission and whose winding down created an occasion for pondering the different forms and functions of bioethics commissions.

The first four essays in this report offer a synoptic, comparative view of national bioethics commissions. A second batch of essays delves more deeply into the goals and mode of operation of specific commissions, and the final group builds on the second by offering more personal reflections about how the commissions did their work and how the work was received. The essayists include commission members, staff directors of commissions, and scholars who have studied the commissions. U.S. commissions are the primary focus, but a few essays look at commissions in other countries; indeed, as Eugenijus Gefenas and Vilma Lukaseviciene note in an essay on efforts to establish commissions in low‐ and middle‐income countries, some bioethical problems “traverse national borders” and need to be taken up collaboratively.

Arras's comment is about the nature of deliberation in a bioethics commission. His lament about paint‐by‐number ethics goes to a seeming choice between richer, deeper moral inquiry and the delineation of more accessible positions; a richer inquiry seems desirable, but outlining broadly accepted if possibly bland positions seems necessary. A related question is about the breadth of inquiry. In an essay about commissions’ choice of topics, Jason Schwartz calls for augmenting the usual focus on technology and research with more discussion of health policy—access to health care and public health, for example. Broader inquiry is in some sense an alternative to richer inquiry, insofar as both going broader and going richer make bigger demands on a commission's available bandwidth. In another sense, broader inquiry might itself benefit from richer inquiry, and indeed, broadening the inquiry might be seen as one way of enriching it: an ethical analysis of access to health care leads, after all, into difficult and contested questions about solidarity, liberty, the importance of core human capacities to human welfare, and the importance of individual responsibility in a well‐functioning society.

Another question about the nature of deliberation has to do with membership in commissions. Many of the essays here touch on this question in one way or another. A presidential commission tends to be an elite group, drawing its members from the ranks of prominent figures in science, law, professional bioethics. Who speaks for the public? Activist organizations who come forward to speak at public meetings of a commission may present themselves as representing the public's concerns, but it's often an open question whether they're right. Reserving a seat or two in a commission for people who bring no relevant professional or disciplinary perspective may ensure that the commission's language does not become too arcane and obscure, but a couple of people can scarcely be said to represent “the public,” and after a few meetings, when they have learned the lingo and really become part of the group, they might, in effect, be elites themselves. Thus, a commission might very nearly be elite by definition.

Gutmann and Wagner argue in this report that “democratic deliberation,” a concept that owes much to Gutmann's own scholarship, was the central theme of the PCSBI's work. Here, they present democratic deliberation as having been achieved through openness—“which involves hearing from diverse people and perspectives, the giving of reasons, and the careful evaluation of evidence.” The moral positions then percolate out of this give‐and‐take—they are reached “inductively,” as Sulmasy puts it in his essay. U.S. commissions have not, however, been designed to do very much to reach out to and actively engage the greater public—to find out, for example, how the public really feels (upon consideration) about risk and uncertainty in the development of genetically modified organisms. But one must start somewhere, especially if there's a deadline for finishing a report. Perhaps, then, the PCSBI's work is best viewed as an example of democratic deliberation but also only as a beginning—a process that should ramify out to various publics as well as to other elite groups. (Along these lines, I once heard Harold Shapiro, chair of the National Bioethics Advisory Commission and a number of other bodies, say that he often told members of these bodies not to take themselves too seriously because whatever they did would be augmented, corrected, and maybe replaced by other bodies.) Perhaps the PCSBI's commitment to public education, as described both by Gutmann and Wagner and by Lisa Lee, could even be seen as a step toward fostering further, later public deliberation.

Yet one more question relevant to Arras's puzzle about the nature of committee deliberation is about the impact that a commission is supposed to have. Many of the essays muse on this theme: Should a commission affect public policy? Public opinion? Scholarship, as measured by scholarly citations to the commission's reports? Or something more complex and less quantifiable—the encouragement of thoughtful attention to certain problems, recognition of their complexity, and perhaps a nudge to move in one direction or another, attending to certain ways of formulating a question or exploring certain distinctions? Different goals might call for different approaches to the deliberation—for research on human subjects, the development of actionable recommendations; but for the prospect of human enhancement, in‐depth, nonconsensus‐based inquiry. In low‐ and middle‐income countries, write Gefenas and Lukaseviciene, national bioethics commissions often have a capacity‐building function: they are supposed to build the ability within that country to do bioethics, perhaps partly by actually taking on some of the bioethics work that needs doing, such as the review of research protocols.

To some degree, however, maybe any commission's work is partly a matter of capacity building. By exploring and developing the language we need for bioethics (the question of richness) and identifying the issues that require deliberation (the question of breadth), commissions both illustrate and launch the deliberation.

